# ELISA-like QDB method to meet the emerging need of Her2 assessment for breast cancer patients

**DOI:** 10.3389/fonc.2023.920698

**Published:** 2023-03-10

**Authors:** Guohua Yu, Yan Lyu, Lei Jiang, Yunjun Wang, Ying Yin, Jiandi Zhang, Maozhou Yang, Fangrong Tang

**Affiliations:** ^1^ Laboratory of Molecular Pathology, Department of Pathology, Affiliated Yantai Yuhuangding Hospital, Qingdao University, Yantai, Shandong, China; ^2^ Yantai Quanticision Diagnostics, Inc., a Division of Quanticision Diagnostics, Inc. (US), Yantai, Shandong, China

**Keywords:** HER2, QDB, FFPE, LOD, HER2-low, ELISA, IHC, T-DXd

## Abstract

Inherent issues of subjectivity and inconsistency have long plagued immunohistochemistry (IHC)-based Her2 assessment, leading to the repeated issuance of guidelines by the American Society of Clinical Oncology/College of American Pathologists (ASCO/CAP) for its standardization for breast cancer patients. Yet, all these efforts may prove insufficient with the advent of Trastuzumab deruxtecan (T-Dxd), a drug with the promise to expand to tumors traditionally defined as Her2 negative (Her2−). In this study, we attempted to address these issues by exploring an ELISA-like quantitative dot blot (QDB) method as an alternative to IHC. The QDB method has been used to measure multiple protein biomarkers including ER, PR, Ki67, and cyclin D1 in breast cancer specimens. Using an independent cohort (cohort 2) of breast cancer formalin-fixed paraffin-embedded (FFPE) specimens, we validated cutoffs developed in cohort 1 (Yu et al., Scientific Reports 2020 10:10502) with overall 100% specificity (95% CI: 100–100) and 97.56% sensitivity (95% CI: 92.68–100) in cohort 2 against standard practice with the dichotomized absolutely quantitated values. Using the limit of detection (LOD) of the QDB method as the putative cutoff point, tumors with no Her2 expression were identified with the number comparable to those of IHC 0. Our results support further evaluation of the QDB method as an alternative to IHC to meet the emerging need of identifying tumors with low Her2 expression (Her2-low) in daily clinical practice.

## Introduction

Her2 (human epidermal growth factor receptor 2), a tyrosine kinase receptor encoded by the *ERBB2* gene, has long been known to be a negative prognostic factor for breast cancer patients. Her2-targeted drugs, including the humanized monoclonal antibody drug trastuzumab and its related antibody–drug conjugate (ADC), have been developed to treat Her2 positive (Her2+) breast cancer patients defined by the American Society of Clinical Oncology/College of American Pathologists (ASCO/CAP). Given its importance, it is no surprise that Her2 assessment of breast cancer specimens has been an indispensable part of daily clinical diagnostics (1–4).

Until now, Her2 protein levels have been assessed primarily with immunohistochemistry (IHC) in daily clinical practice (4). The updated algorithm of the ASCO/CAP guideline for Her2 assessment consists of first-line assessment of Her2 levels with IHC to classify the cancer specimens into four categories: 0/1+/2+/3+ or IHC 0/IHC 1+/IHC 2+/IHC 3+, with 0/1+ being Her2 negative (Her2−) and 3+ being Her2 positive (Her2+). The 2+ group, as Her2 equivocal, requires further fluorescence *in situ* hybridization (FISH) analysis to identify FISH-positive from FISH-negative patients (4). Accordingly, Her2− includes 0/1+ and 2+/FISH negative, while Her2+ includes 3+ and 2+/FISH positive.

The IHC method is known to be semi-quantitative with inherent subjectivity and inconsistency. After three consecutive ASCO/CAP guideline being issued over more than a decade, various issues remain to be addressed to improve the accuracy and consistency of the IHC results (2–4). These issues become even more problematic with local laboratories and laboratories in developing countries (5). Alternative methods were extensively explored to circumvent these limitations, yet none of them have gained wide acceptance in daily clinical practice for various reasons (6–10). Thus, although FISH only measures DNA amplification as an indirect indication of protein overexpression, it is still considered the gold standard in the field.

Yet, the IHC method faces even more challenges with the advent of Trastuzumab deruxtecan (T-Dxd) (11–13). T-Dxd is a new ADC designed to deliver the chemotherapeutic agent topoisomerase I inhibitor directly to malignant cells. Recent studies demonstrated the anti-tumor activity of T-Dxd even among breast cancer with low Her2 expression, or Her2-low, to include tumors of both 1+ and 2+/FISH negative (14, 15). It should be noted that in these studies, Her2-0 patients were excluded. The suitability of Trastuzumab-emtansine (T-DM1) for Her2-low patients was also actively explored, with and without the presence of CDK4/6 inhibitors (16, 17).

It is not surprising that the IHC-based method is inefficient to separate Her2 1+ from IHC 0 specimens, as “IHC was never developed as a quantitative assay to measure levels of protein expression” (12). As stated in the most recent ASCO/CAP guidelines, IHC 0 is defined as “no staining or faint or residual membranous reactivity in ≤10% of tumor cells” while 1+ is defined as “faint or residual membranous reactivity in >10% of tumor cells” (4). The marginal difference between IHC 0 and 1+ may be too hard to be appreciated by visual evaluation. Software-assisted image analysis may help to address this limitation; however, major efforts may be needed to achieve its standardization in daily clinical practice (18, 19). On the other hand, there is also little effort from oncologists to differentiate IHC 0 from 1+ in daily clinical practice, as both are considered Her2− based on the current guidance.

Even though it may be theoretically achievable to separate IHC 0 from 1+ in daily clinical practice, it would be difficult to establish a fine cutoff to differentiate IHC 0 from 1+ (12). Collective efforts may be needed before an acceptable guidance may be developed in the future to identify Her2-low patients. Furthermore, the concept of “Her2 ultra-low” has also been pursued in a recent clinical trial (DESTINY-Breast06, NCT04494425), where the benefit of T-Dxd will be evaluated on patients with an IHC score between 0 and 1+ (>IHC 0 and<IHC 1). Clearly, alternative methods to measure Her2 levels consistently and quantitatively are urgently needed to meet the pressing need of T-Dxd and its expected peers in the near future.

Recently, we introduced an ELISA-like immunoassay, the quantitative dot blot (QDB) method, to measure protein biomarker levels absolutely and quantitatively in formalin-fixed paraffin-embedded (FFPE) specimens. The QDB method shares a lot of features with ELISA, including being high throughput, objective, quantitative, and easy to perform. Yet, unlike ELISA, this method is able to measure antigen levels in FFPE specimens (20–23). Thus, we considered it as an ELISA-like assay. The method was explored in 332 FFPE breast cancer specimens (cohort 1) using both EP3 and 4B5 antibodies (20). When dichotomously converted, it achieved 99.5% concordance with IHC and 94.2% concordance with FISH, with overall concordance at 96.9% (20).

With the potential expansion of T-Dxd to all but those of IHC 0 patients, we interpreted that the ongoing efforts were aimed at differentiating tumors with no Her2 expression (Her2-0) from tumors with any Her2 expression (Her2-E). In this study, we validated the QDB method in an independent cohort (cohort 2) of breast cancer specimens using cutoffs developed in cohort 1 to evaluate its performance against current standard IHC/FISH protocol. The feasibility of its limit of detection (LOD) to differentiate Her2-0 from Her2-E in daily clinical practice was also explored as an option to meet the emerging need of T-Dxd and its expected peers for breast cancer patients.

## Materials and methods

### Human subjects

Two independent cohorts were used in this retrospective study. The inclusion criteria for this retrospective observational study were female patients diagnosed as breast cancer with FFPE tissue available at Yuhuangding Hospital at Yantai, China. For cohort 1, a total of 332 FFPE breast cancer specimens were collected as 2 × 5 μm slices from patients admitted from January 2015 to August 2017 sequentially and non-selectively as described previously (20). For cohort 2, a total of 246 FFPE breast cancer specimens were collected sequentially and non-selectively as 2 × 15 μm slices from patients admitted from 5 January to 30 December 2010 as described elsewhere (24). While 2 × 5 μm slices from each specimen were more than sufficient for Her2 assessment using the QDB method, more tissue slices were requested from cohort 2 to study other protein biomarkers (24). For both studies, a minimum of 50% tumor tissue is required based on H&E staining of the provided slices. All the studies were carried out in accordance with the Declaration of Helsinki, and approved by the Ethics Committee of Yuhuangding Hospital ([2017]76 to Guohua Yu) with an informed consent waiver due to the use of archival tissues with retrospective, anonymized clinical data. The cellular controls were described previously (20). All IHC analyses were performed using the 4B5 antibody, and the results were documented in patients’ medical records.

### General reagents

All of the chemicals were listed previously, including the detailed procedures to prepare cellular controls (20). In brief, recombinant human HER2/ErbB2 protein was purchased from Sino Biological Inc. Ventana anti-HER2/neu (4B5) rabbit monoclonal primary antibody was purchased from Roche Diagnostics GmbH. Rabbit anti-HER2 monoclonal antibody (clone EP3) was purchased from ZSGB-BIO. The QDB plate was provided by Quanticision Diagnostics, Inc. (RTP, USA).

### QDB analysis

The preparation of FFPE lysates has been described in detail previously (20, 22). In brief, two FFPE tissue slices (5 µm for cohort 1 or 15 μm for cohort 2) were de-paraffinized before solubilization using lysis buffer (50 mM HEPES, 137 mM NaCl, 5 mM EDTA, 1 mM MgCl_2_, 10 mM Na_2_P_2_O_7_, 1% Triton X-100, and 10% glycerol). The supernatants were collected after centrifugation at 21,000*g* for 15 min, and the total amount of protein was determined using the BCA protein assay kit (Thermo Fish Scientific, Waltham, MA).

**Figure 1:**
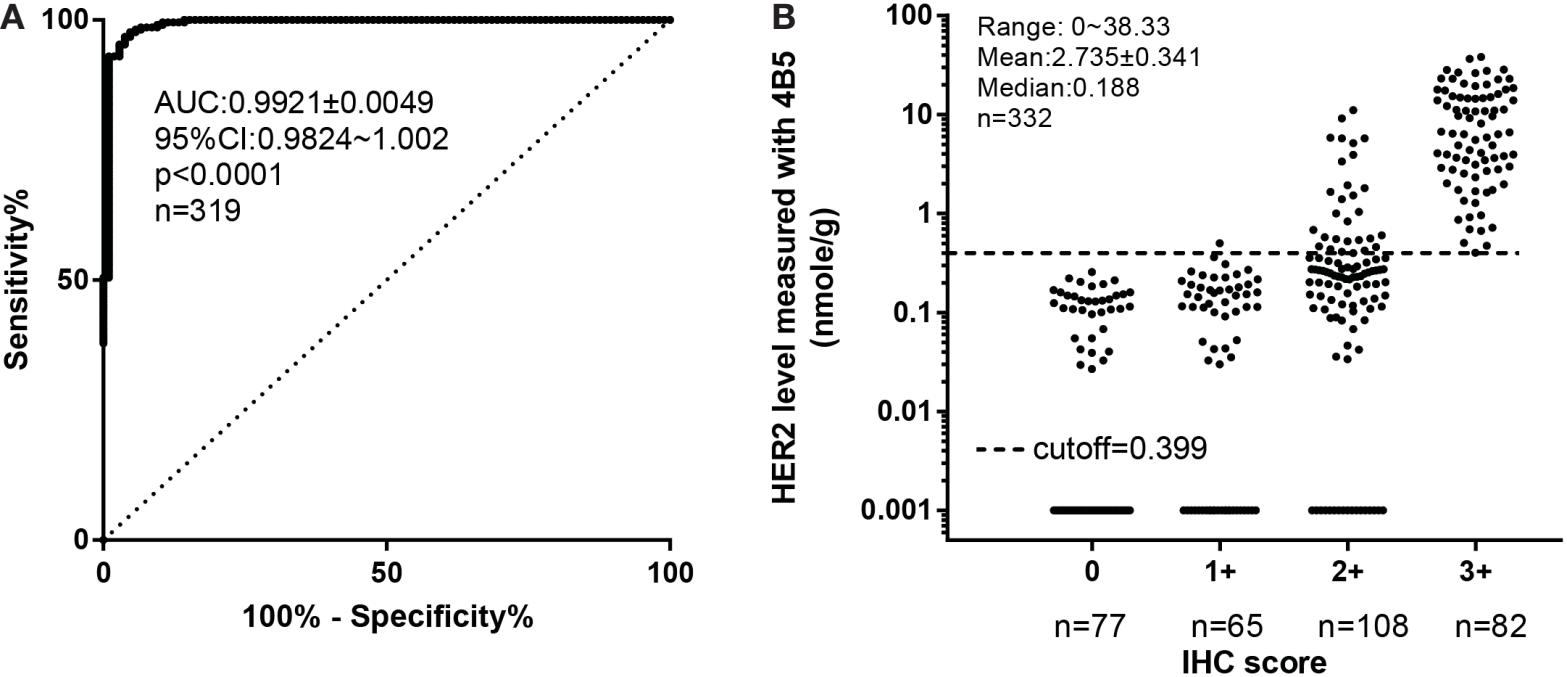
Defining the optimized cutoff for 4B5 using receiver operating characteristic (ROC) analysis based on the standard protocol of FISH/IHC analyses. **(A)** ROC analysis of QDB results. **(B)** The distribution of HER2 levels within each IHC category. All the FFPE specimens in cohort 1 were categorized as Her2+ and Her2− based on combined IHC and FISH analyses. FISH results superseded the IHC results whenever a conflict occurred. Her2 levels measured by the QDB method using the 4B5 antibody were plotted against categorized Her2 results for ROC analysis (*n* = 319) in **(A)**. In **(B)**, Her2 levels measured with QDB were plotted against each IHC categorized scores. Log scale was used to illustrate the performance of the proposed cutoff at 0.399 nmol/g. For specimens with undetectable Her2 levels, a value at 0.001 nmol/g was arbitrarily entered to avoid missing any specimen in the graph.

The process of QDB assay was illustrated in detail in **Figure 1** of a previous study (20). The final concentration of FFPE tissue lysates was adjusted to 0.25 μg/μl, and loaded onto the QDB plate at 0.5 μg/unit in triplicate. The plates were dried at room temperature for 4 h before they were blocked in blocking buffer [4% non-fat milk in Tris-buffered saline with 0.1% Tween 20 (TBST)] for another hour. The plates were incubated either with EP3 at 1:1500 in blocking buffer or with 4B5 at 1:10 in PBS overnight. After incubating with an HRP-labeled donkey-anti-rabbit secondary antibody for another 3 h, the plates were rinsed with TBST, and washed with TBST 5 × 10 min before they were inserted into a white 96-well plate pre-filled with 100 μl/well ECL working solution for 3 min. The plates were quantified using a Tecan Infinite 200PRO Microplate reader with the option “plate with cover”. BT474 and MCF-7 cell lysates with known HER2 level were included in all the experiments as internal controls to ensure the consistency of the results.

### Defining the limit of detection of the QDB method

The LOD was defined as the concentration value derived from the mean value of 18 zero standard replicates plus 2 standard deviations (SD). The value was further converted into an absolute value using recombinant Her2 protein. The mean LOD was established from 10 independent assays.


(1) $$LOD=\frac{\sum_{i=1}^{18}x_{i}}{18}+2SD_{s}$$

(2) $$mean\;LOD=\frac{\sum_{k=1}^{10}LOD_{k}}{10}$$

For QDB-based Her2 assessment using both 4B5 and EP3 antibodies, the LODs were all calculated at 0.09 nmol/g.

### IHC analysis

All the IHC analyses were performed as a routine clinical practice by a certified pathologist in the hospital by strictly following the guidance issued by ASCO/CAP (2–4). Briefly, IHC for Her2 was performed with the standard streptavidin–biotin complex method with 3,3’-diaminobenzidine as the chromogen. PATHWAY anti-Her2/neu (4B5) Rabbit Monoclonal Primary Antibody (Ventana Medical Systems, Inc.) was used for all the analysis using the Ventana Benchmark XT system by following the manufacturer’s instructions.

### FISH analysis

For cohort 1, FISH results were primarily based on documented medical records (*n* = 128). The specimens with discordant QDB/FISH results (*n* = 16) were submitted to ZSGB-Bio, Inc. (https://www.zsbio.com) for independent validation. For cohort 2, a total of 19 samples with discordant QDB/IHC results were submitted to ZSGB-Bio, Inc. for FISH analysis. The detailed reports are available upon request.

For all FISH analysis, a breast cancer Her2 detection kit (FISH method) manufactured by Guangzhou LBP Medicine Science and Technology Co., Ltd. was used following the manufacturer’s instructions. The kit contains a HER2/neu probe and a chromosome 17 centromere (*CEP17*) probe. The absolute *HER2* signal and the ratio of *HER2/CEP17* signals were reported, and Her2 status was assessed by strictly following the guidance of ASCO/CAP.

### Statistical analysis

All statistical analyses were performed using GraphPad Prism software version 7.0 (GraphPad Software Inc., USA) and R 4.0.1(http://www.r-project.org). Her2 positive and Her2 negative were defined by following the ASCO/CAP guidance or with the quantitated Her2 levels above (Her2 positive) or below (Her2 negative) the proposed cutoffs of 0.399 nmol/g for 4B5 or 0.261 nmol/g for EP3. ROC analysis was used to determine the optimized cutoffs for QDB_4B5_ and QDB_EP3_, respectively. Continuous variables were expressed as mean ± SEM. The correlation analysis was performed using either Pearson’s correlation coefficient analysis or Spearman’s rank correlation analysis as specified in the manuscript. The sensitivity, specificity, diagnostic accuracy, PPV, and NPV were calculated for QDB_4B5_ and QDB_EP3_. 95% CI was calculated using the “ci.coords” function from the “pROC” R package. One specimen was excluded from all QDB measurements using the 4B5 antibody due to the insufficient amount of extracted protein. Specimens missing IHC and/or FISH results were also excluded in the corresponding studies as specified in the manuscript. A *p*-value of<0.05 was considered statistically significant for all tests.

## Results

### Developing putative cutoff for the 4B5 antibody

The clinicopathological characteristics of the 332 FFPE breast cancer specimens in cohort 1 have been reported previously (20). Her2 protein levels measured with the 4B5 antibody in cohort 1 were used to develop its optimized cutoff based on the combined results of IHC and FISH analyses (IHC/FISH, including IHC 0, IHC 1, IHC 2+ with FISH results, IHC3, and specimens with discordant FISH/QDB results further validated with independent FISH analysis). As shown in **Figure 1A**, Her2 levels were highly correlated with results from IHC/FISH, with the area under ROC curve (AUC) from ROC analysis at 0.9921 ± 0.0049, *p*< 0.0001. At a cutoff of 0.399 nmol/g, we achieved 96.73% specificity (95% CI: 92.06%–99.53%) and 96.19% sensitivity (95% CI: 93.33%–100%). When Her2 levels from cohort 1 were converted dichotomously, the concordance rate was 99.6% between QDB and IHC (**Figure 1B**), 92.8% between QDB and FISH (*n* = 144), and 96.6% when concordance was calculated with IHC/FISH. In cohort 1, the concordance rate between Her2 levels measured with 4B5 and EP3 was 98.8% (**Supplementary Table S1**).

### Validating cutoffs developed in cohort 1 and cohort 2

**Figure 2:**
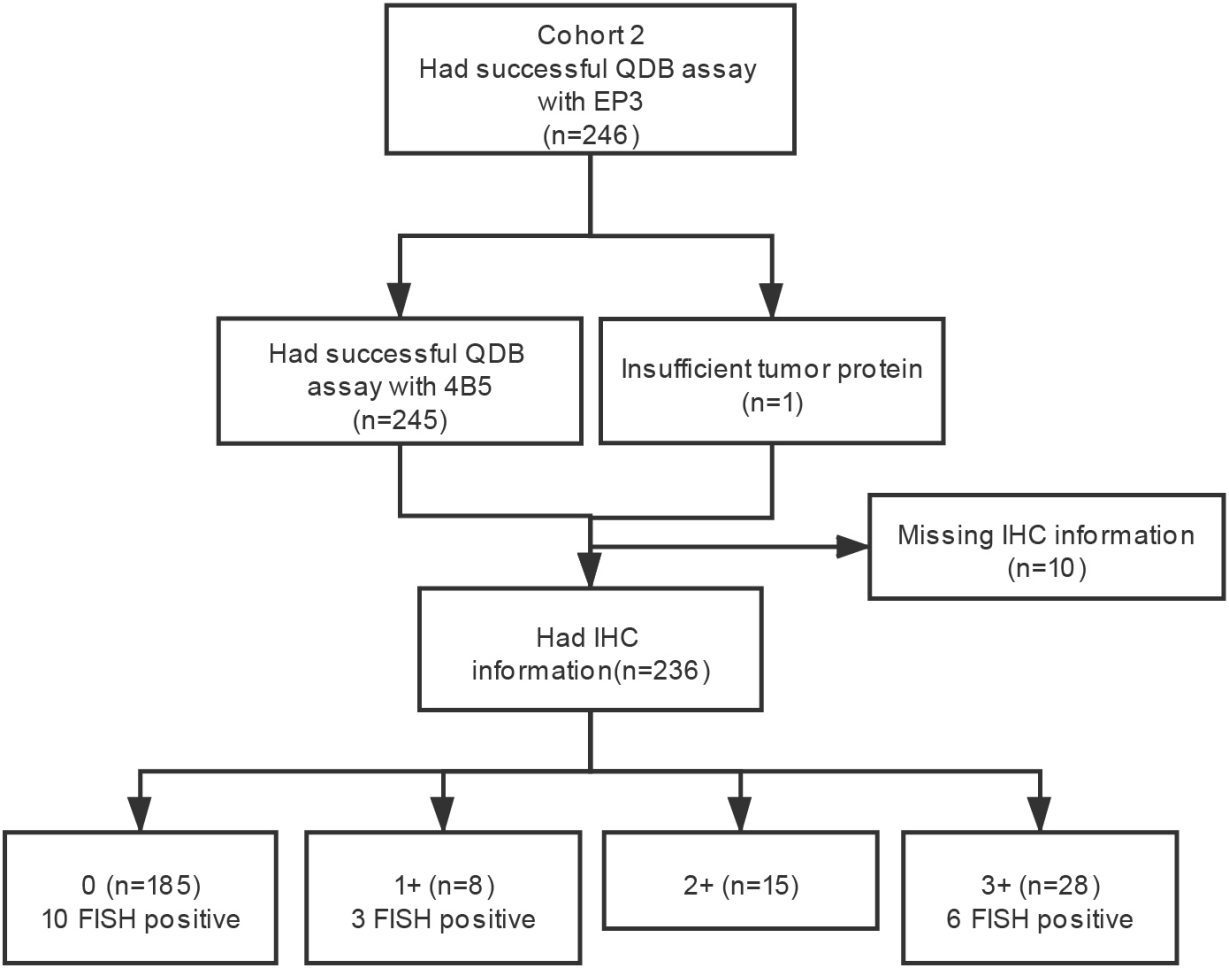
Flow diagram of patient selection for the study in cohort 2.

**Figure 3:**
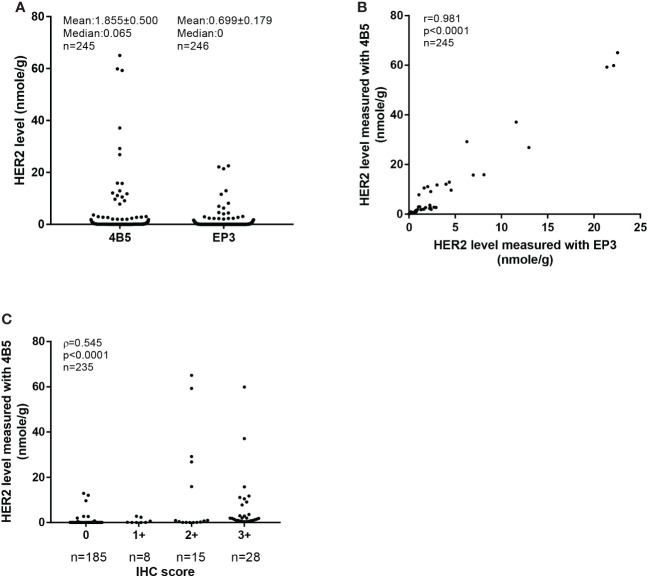
Absolutely quantitative analysis of Her2 expressions in cohort 2. **(A)** The distribution of HER2 levels measured with the QDB method using 4B5 and EP3 antibodies respectively in cohort 2. The results were expressed as mean ± SEM. **(B)** Correlation analysis of the Her2 levels in cohort 2 measured with the QDB method using 4B5 and EP3 antibodies, respectively. Pearson’s correlation analysis was performed with *r* = 0.981, *p*< 0.0001. **(C)** Correlation of HER2 levels measured with QDB using the 4B5 antibody with those from IHC analysis. The results were analyzed using Spearman’s correlation analysis with ρ = 0.545, *p*< 0.0001.

The clinicopathological characteristics of 246 FFPE breast cancer specimens from cohort 2 were described previously (22). Based on the provided IHC results, there were 185 specimens categorized as IHC 0, 8 categorized as 1+, 15 categorized as 2+, and 28 categorized as 3+ (**Figure 2**). The distribution of the Her2 protein levels measured with the 4B5 antibody was from 0 (undetectable) to 65.05 nmol/g, and 0 to 26.58 nmol/g for those measured with the EP3 antibody (**Figure 3A**). The measured Her2 levels with 4B5 and EP3 were highly related, with *r* = 0.981, *p*< 0.0001 based on Pearson’s correlation analysis (**Figure 3B**). The absolutely quantitated Her2 levels with 4B5 and EP3 were also highly correlated with IHC results when analyzed with Spearman’s correlation analysis, with *ρ* = 0.545, *p*< 0.0001 and *ρ* = 0.651, *p*< 0.0001 respectively (**Figure 3C** and **Supplementary Figure 1**). To avoid confusion, we limited our discussion to QDB measurements with 4B5 in most cases, and results with EP3 were only discussed when specified.

The absolutely quantitated Her2 levels in cohort 2 were converted into Her2+/Her2− using the cutoff developed in cohort 1. Accordingly, there were 195 Her2− and 50 Her2+ identified in cohort 2. The concordance rate between QDB and IHC was 93.2% (**Supplementary Table S2**).

### Evaluation of discordant specimens with FISH

A total of 19 discordant specimens were identified between IHC and QDB using the EP3 antibody in cohort 2. Surprisingly, among these 19 specimens, we only identified 14 discordant specimens using the 4B5 antibody (13 specimens of IHC 0/1+ and one specimen of 3+), with the other five discordant specimens (all 3+ specimens) being EP3 specific. It should be mentioned that all the IHC analyses were performed using the 4B5 antibody. All these discordant specimens were sent out and identified as Her2 positive from FISH analysis by a third party. Accordingly, we rescued 13 Her2-positive specimens from 0/1+ groups and missed one Her2-positive specimen with the QDB method using the 4B5 antibody. Thus, QDB achieved 92.9% agreement with FISH among 14 discordant specimens with the 4B5 antibody. Our data also indicated that that there was a subtle difference between the 4B5 and EP3 antibodies in detecting Her2 protein in breast cancer specimens.

### Evaluation of the performance of the QDB method in the merged cohort

**Figure 4:**
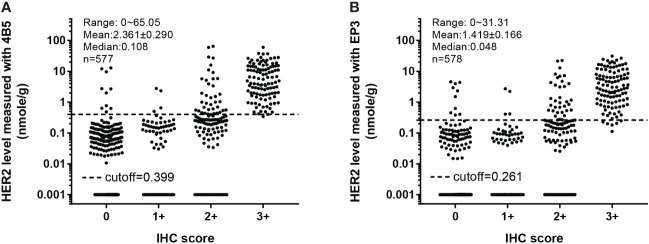
Overall distribution of absolutely quantitated HER2 levels in the merged cohort within each IHC category. The Her2 protein levels in both cohort 1 and cohort 2 were measured with the QDB method using EP3 and 4B5 antibodies, respectively, and plotted together against their respective IHC scores. **(A)** In QDB analysis with the 4B5 antibody, Her2 levels ranged from 0 to 65.05 nmol/g, with a mean of 2.361 ± 0.290 and a median of 0.108, *n* = 577. **(B)** For those with the EP3 antibody, its levels ranged from 0 to 31.31 nmol/g, with a mean of 1.419 ± 0.166 and a median of 0.048, *n* = 578.

One advantage of the QDB assay is that different cohort studies can be merged together as Her2 levels were measured as absolute values. We integrated cohort 1 (*n* = 332) and cohort 2 (*n* = 246) into a single group, and showed the distribution of absolutely quantitated Her2 protein levels of the merged cohort (*n* = 578) measured with 4B5 and EP3 antibodies, respectively (**Figure 4**).

When converting QDB results dichotomously, we achieved overall concordance using EP3 and 4B5 at 98.3% (**Table 1**). We achieved sensitivity and specificity at 96.58% (95% CI: 93.15%–99.32%) and 98.22% (95% CI: 96.69%–99.24%) for 4B5, and 93.84% (95% CI:89.73%–97.26%) and 98.22% (95% CI: 96.69%–99.24%) for EP3, in contrast to the 100% (95%CI: 85.1%-100%) and 66.7% (95% CI: 51.0% -79.4%) of IHC (**Table 2**). It should be noted that there might be a bias in the analysis, as we only sent discordant specimens for FISH analysis.

**Table 1:** Comparison of dichotomized QDB results measured with 4B5 and EP3 using their respective cutoffs in the merged cohort (cohorts 1 and 2; *n* = 578).

		With the 4B5 antibody			
		HER2−	HER2+	Undetermined	Total
With the EP3 antibody^*^	HER2−	414	8	1	423
	HER2+	2	153	0	155
	Total	416	161	1	578

Concordance rate: 98.3%.

*Based on the cutoff value of 0.261 nmol/g for EP3 and 0.399 nmol/g for 4B5. All the 578 samples were rated as Her2+ or Her2− based on the proposed cutoff value of 0.261 nmol/g for QDB_EP3_ and 0.399 nmol/g for QDB_4B5_. The concordance rate of QDB results was calculated as (414 + 153)/(416 + 161) × 100% = 98.3%.

**Table 2:** Sensitivity and specificity of Her2 levels from the QDB method using 4B5 and EP3 antibodies, respectively, vs. IHC (*n* = 578) using FISH results as the gold standard.

	Specificity % (95% CI)	Sensitivity % (95% CI)	Accuracy %	NPV %	PPV %
4B5	98.22 (96.69–99.49)	96.58 (93.15–99.32)	97.77	98.72	95.27
EP3	98.22 (96.69–99.24)	93.84 (89.73–97.26)	97.03	97.72	95.14
IHC	66.7 (51–79.4)	100 (85.1–100)	62.86	100	78.69

PPV, positive predicted value; NPV, negative predictive value.

### Evaluating the LOD of the QDB method to differentiate specimens with no Her2 expression (Her2-0) from specimens with any Her2 expression

We also evaluated the LOD of the QDB method to differentiate Her2-0 from Her2-E with these two breast cancer cohorts. The LOD of the QDB assay was calculated at 0.09 nmol/g with the 4B5 antibody. Using this value as a putative cutoff point, we were able to stratify breast cancer specimens into Her2-0 and Her2-E groups. Next, the performance of the QDB method was compared with that of IHC in **Table 3**. We were able to identify 64.9%, 47.7%, 24.1%, and 0% Her2-0 specimens in cohort 1, and 78.9%, 50%, 33.3% and 0% Her2-0 specimens in cohort 2 in IHC 0, 1+, 2+, and 3+ groups, respectively. In the merged cohort, there were 74.8%, 47.9%, 25.2%, and 0% Her2-0 among IHC 0, 1+, 2+, and 3+ groups, respectively. The overall concordances between QDB and IHC were calculated at 76.9%.

**Table 3:** Evaluation of the distribution of breast cancer specimens with no Her2 expression (Her2-0) from those with any Her2 expression (Her2-E) in each IHC category based on the limit of detection (LOD) of the QDB method using 4B5 antibodies.

	Cohort1			Cohort2			Merged		
	-	+	Total	-	+	Total	-	+	Total
**0**	50 (64.94%)	27 (35.06%)	77	146 (78.92%)	38 (20.54%)	185	196 (74.81%)	65 (24.81%)	262
**1+**	31 (47.69%)	34 (52.31%)	65	4 (50.00%)	4 (50.00%)	8	35 (47.95%)	38 (52.05%)	73
**2+**	26 (24.07%)	82 (75.93%)	108	5 (33.33%)	10 (66.67%)	15	31 (25.20%)	92 (74.80%)	123
**3+**	0 (0.00%)	82 (100.00%)	82	0 (0.00%)	28 (100.00%)	28	0 (0.00%)	110 (100.00%)	110
**Total**	107 (32.23%)	225 (67.77%)	332	155 (65.96%)	80 (34.04%)	235	262 (46.21%)	305 (53.79%)	567

We also evaluated the consistency of the QDB method to stratify Her2-0 from Her2-E using LODs from EP3 and 4B5, respectively. Coincidentally, the LOD of the QDB method using the EP3 antibody was also at 0.09 nmol/g. When results from the EP3 antibody were dichotomized using its LOD, we identified 339 cases of Her2-0 vs. 239 of Her2-E, in comparison to 207 of Her2-0 vs. 307 Her2-E from the 4B5 antibody. The overall concordance rate was 85.3% (**Supplementary Table S3**).

The consistency of the results was also examined, as shown in **Supplementary Table S4**. When the same group of specimens were analyzed by different scientists, their concordance rates were all above 90% among each other.

## Discussion

In this study, we used two independent cohorts of breast cancer specimens to demonstrate the suitability of an ELISA-like immunoassay, the QDB method, for absolute quantitation of Her2 protein levels to meet the need of daily clinical practice. Importantly, our results suggested that it may provide a new option to identify Her2-low patients, which may benefit from T-Dxd and its expected peers in daily clinical practice.

Intensive efforts have been devoted to developing a feasible method to identify Her2-low patients in daily clinical practice ever since the advent of T-Dxd. There are mainly two directions to be explored to address this pressing need. One direction is to improve the consistency and accuracy of the existing IHC method, either by more rigorous and better-defined guidelines (25) or through software-assisted image analysis (18, 26). The other direction is to measure Her2 biochemically at the cost of tissue morphology. A method of immunoaffinity enrichment coupled with mass spectrometry (MS) was developed with impressive results (27). Yet, both these two directions have their limitations. The IHC method was not developed as a quantitative assay (12). The 0/1+/2+/3+ would set artificial cutoffs to stratify patients without any clinical basis. That also explains why current efforts are devoted to identifying “Her2 ultra-low” (Her2 levels between IHC 0 and IHC 1) in DESTINY-Breast06 trial (NCT04494425). The MS method would provide a satisfied solution to identify both Her2-low and Her2 ultra-low patients with its unmatched sensitivity. However, the complicated analytical process makes it an unlikely choice in daily clinical practice.

The QDB method provides a more feasible choice for daily clinical practice in comparison to both software-assisted image analysis and MS. Like ELISA, this method is much easier to be standardized, requiring minimum equipment and technical skills to be performed in a typical clinical laboratory setting. Currently, intensive efforts have been devoted to seeking regulatory approval of QDB-based Her2 assay worldwide.

Using two well-established IVD-grade antibodies (EP3 and 4B5), we measured Her2 levels in cohort 1 (*n* = 332) and cohort 2 (*n* = 246) absolutely, quantitatively, and objectively. Based on the cutoff values developed in cohort 1, Her2 levels were converted dichotomously to achieve an overall concordance of 97.6% with IHC/FISH in the merged cohort.

The QDB method was demonstrated here to offer several advantages at the cost of the context of tissue morphology. First, it provided more reliable and consistent assessment than IHC for both cohorts of breast cancer specimens. Second, the implementation of the QDB platform in clinical practice may render unnecessary FISH analysis for all the IHC equivocal cases, as QDB analysis can effectively separate Her2+ from Her2− specimens by dichotomously converting the measured Her2 levels using a pre-determined cutoff. In this study, the concordance between QDB and FISH was at 93.0% (*n* = 163) (**Supplementary Table S5**). Finally, it is the only feasible choice to provide absolutely quantitated Her2 levels in daily clinical practice. The only other method is MS (6, 7, 28). However, the high complexity of the technique and its low-throughput nature have limited its implementation in daily clinical practice. Our current studies warrant future prospective studies of QDB-based Her2 assessment to meet regulatory requirements for its implementation in daily clinical practice.

In addition, like all ELISA-like immunoassays, the LOD of the QDB method may be considered as a feasible cutoff point to distinguish tumors with no Her2 expression from the rest of the population. This is especially important considering the inability of the IHC method to distinguish IHC scores of 0 from 1+ and the ongoing efforts to identify “Her2 ultra-low” (DESTINY-Breast06, NCT04494425). Clearly, the current standard practice is insufficient to meet the emerging need of T-Dxd and its expected peers, which may potentially progress as a first-line treatment for breast cancer patients in the near future (17).

**Figure 5:**
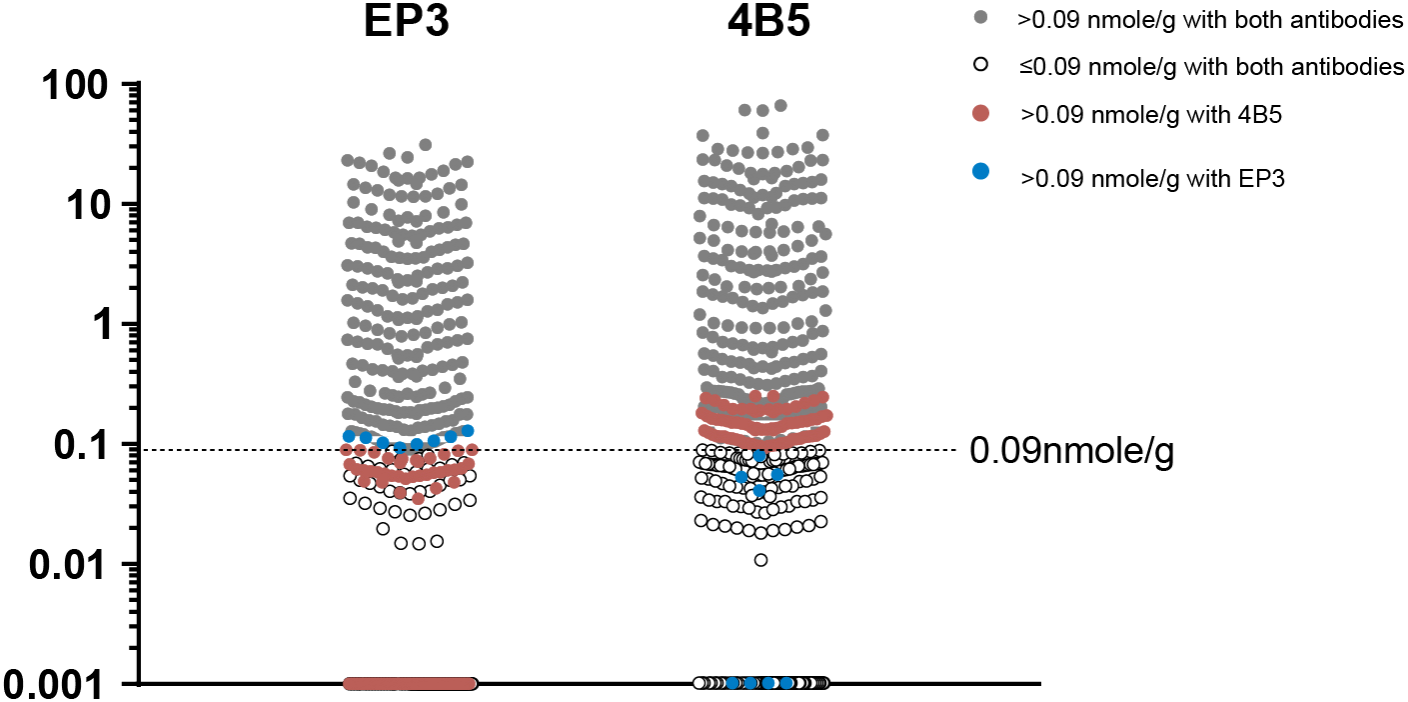
The distribution of specimens with (Her2-E) and without Her2 expression (Her2-0) stratified by limit of detection (LOD) of QDB analysis using either EP3 or 4B5 antibodies. The LODs of QDB analysis with both 4B5 and EP3 antibodies were at 0.09 nmol/g. Specimens with Her2 levels above 0.09 nmol/g with both antibodies were in gray color while those below 0.09 nmol/g were in the white circle. Specimens with Her2 levels above 0.09 nmol/g only when measured using the 4B5 antibody were in brown color while those only with the EP3 antibody were in blue color.

We achieved 85.3% concordance when using the LOD of both EP3 and 4B5 antibodies as the cutoff to identify the putative Her2-0 group. We interpreted that this difference was from antibodies rather than from the method *per se*. The QDB results from EP3 were clearly in a narrower range than those from 4B5 (0–31.31 nmol/g vs. 0–65.05 nmol/g). Yet, the LODs for both antibodies were at 0.09 nmol/g, which inevitably leads to more Her2-0 (*n* = 339) from EP3 than those from 4B5 (*n* = 270). In fact, we observed that a fair portion of discordant specimens were in close proximity to the LOD of EP3 (**Figure 5**). Thus, more specimens were categorized as Her2-0 by EP3 due to a relatively higher cutoff value in reference to its narrower expression range. Therefore, the number of presumed Her2-0 cases may be heavily influenced by the antibody used in the analysis. It is difficult at the current stage to judge which antibody would be more relevant in clinical practice. Outcome-based analysis is needed to find the right one for this purpose.

We were unable to explain the rare case where a specimen was identified as Her2− by QDB using both 4B5 and EP3 antibodies, but as Her2+ by both IHC and FISH. We expect the results from the QDB method to match 100% with those from an ideally performed IHC analysis, when the IHC result is no longer plagued by the subjectivity issue and tumor heterogeneity. However, this lone exception hinted that there are always rare cases (1/578) warranting further exploration in the future.

## Conclusions

In summary, through studies of two independent cohorts of breast cancer FFPE specimens, we established the QDB method as a more accurate and reliable method than IHC in assessing Her2 protein levels. We also validated the antibody-specific cutoffs to convert absolutely quantitated Her2 protein levels into conventional dichotomous Her2+/Her2− for routine clinical use. Additionally, our results strongly suggested that the LOD of this method might provide a putative cutoff point to identify breast tumors with no Her2 expression, bypassing the difficulty of using IHC to define the Her2-low subgroup of breast tumors. It warrants further exploration in the near future as a proposed companion diagnostic test to identify Her2-low breast cancer patients for T-Dxd treatment.

## Data availability statement

The raw data supporting the conclusions of this article will be made available by the authors, without undue reservation.

## Ethics statement

The studies involving human participants were reviewed and approved by Ethics committee of Yuhuangding Hospital (Approval #: [2017]76). Written informed consent for participation was not required for this study in accordance with the national legislation and the institutional requirements.

## Author contributions

GY, LJ, YW, and YY provided clinical samples and performed IHC analyses; GY supervised all the clinical studies; YL performed all the statistical analysis, YL and FT performed all the assays and performed data analysis; JZ designed and supervised the overall study and drafted the manuscript; GY, JZ, MY, and FT contributed to data interpretation and edited the manuscript. All authors contributed to the article and approved the submitted version.
