# Supplementary material for: ELISA-like QDB method to meet the emerging need of Her2 assessment for breast cancer patients
Source: Front Oncol. 2023 Mar 10;13:920698. doi: 10.3389/fonc.2023.920698 (PMC10036774; doi:10.3389/fonc.2023.920698)
Supplement: Supplementary file 1 [file DataSheet_1.docx]

**Supplemental Figures**


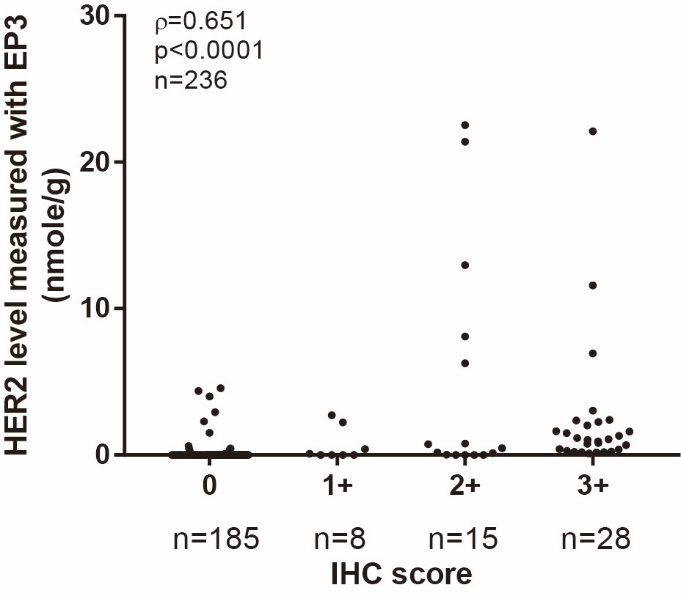


**Figure S1:** Correlation of HER2 levels from QDB method using EP3 antibody with those from IHC analysis using 4B5 antibody. The results were analyzed using Spearman’s correlation analysis with r=0.651, p<0.0001.

**Supplemental Tables**

**Table S1:** Concordance of QDB results measured with 4B5 and EP3 antibodies respectively in cohort 1

|  |  |  | With 4B5 ^*^ | |  |
| --- | --- | --- | --- | --- | --- |
|  |  |  | HER2 - | HER2 + | Total |
| With EP3 ^*^ | HER2 - |  | 219 | 2 | 221 |
|  | HER2 + |  | 2 | 109 | 111 |
|  | Total |  | 221 | 111 | 332 |

Concordance rate: 98.8%

*Based on cutoff values at 0.261 nmole/g for EP3, and 0.399 nmole/g for 4B5 respectively.

All 332 samples were rated as Her2- or Her2+ based on the proposed cutoff value at 0.261 nmole/g for EP3 and 0.399 nmole/g for 4B5, and for description purpose, labeled collectively as QDB4B5 and QDBEP3 based on the antibody used in the analysis. The concordance rate of QDB was calculated as (219+109)/332×100%=98.8%.

**Table S2:** Concordance of Her2 levels from QDB method with those of IHC

|  |  | QDB^*^ | | |  |
| --- | --- | --- | --- | --- | --- |
|  |  | HER2- | HER2+ | Unknown | Total |
| IHC^#^ | 0 | 174 | 10 | 1 | 185 |
|  | 1 | 5 | 3 | 0 | 8 |
|  | 2 | 6 | 9 | 0 | 15 |
|  | 3 | 1 | 27 | 0 | 28 |
|  | Unknown | 9 | 1 | 0 | 10 |
|  | Total | 195 | 50 | 1 | 246 |

The concordance rate was 93.2% between QDB and IHC.

*Based on cutoff value at 0.399 nmole/g for 4B5.

#: Specimens of 2+ from IHC analysis were excluded from analysis.

The concordance rate of QDB and IHC was calculated as (174+5+27)/(185+8+28)×100%=93.2%.

**Table S3:** Concordance of specimens with (Her2-E) and without Her2 expression (Her2-0) based on the Limit of Detection (LOD) of the QDB method using 4B5 and EP3 antibodies respectively.

|  | | | EP3 | |  |
| --- | --- | --- | --- | --- | --- |
|  |  |  | - | + | total |
| 4B5 | cohort1 | - | 100 | 7 | 107 |
|  |  | + | 57 | 168 | 225 |
|  |  | unknown | 0 | 0 | 0 |
|  |  | total | 157 | 175 | 332 |
|  | cohort2 | - | 162 | 1 | 163 |
|  |  | + | 20 | 62 | 82 |
|  |  | unknown | 0 | 1 | 1 |
|  |  | total | 182 | 64 | 246 |
|  | merge | - | 262 | 8 | 270 |
|  |  | + | 77 | 230 | 307 |
|  |  | unknown | 0 | 1 | 1 |
|  |  | total | 339 | 239 | 578 |

The concordance rate was calculated as (262+230)/577= 85.3%

**Table S4:** The consistency of the QDB analysis obtained from three scientists. The same group of specimens were measured side by side by two different scientists respectively, and their concordance rate was calculated using 0.09 nmole/g as cutoff.

|  | | Scientist-2 | | |
| --- | --- | --- | --- | --- |
|  |  | <0.09 nmol/g | ≥0.09 nmol/g | Total |
| Scientist-1 | <0.09 nmol/g | 182 | 0 | 196 |
|  | ≥0.09 nmol/g | 14 | 50 | 50 |
|  | Total | 182 | 64 | 246 |

Concordance rate: 94.3%.

|  | | Scientist-3 | | |
| --- | --- | --- | --- | --- |
|  |  | <0.09 nmol/g | ≥0.09 nmol/g | Total |
| Scientist-1 | <0.09 nmol/g | 265 | 19 | 284 |
|  | ≥0.09 nmol/g | 7 | 70 | 77 |
|  | Total | 272 | 89 | 361 |

Concordance rate: 92.8%.

**Table S5**: Concordance of QDB_4B5_ with FISH results in merged cohorts of 1 and 2

|  | **QDB_4B5_*** | | | |
| --- | --- | --- | --- | --- |
|  |  | **Her2-** | **Her2+** | **Total** |
| **FISH^#^** | **Her2-** | 89 | 6 | 95 |
|  | **Her2+** | 5 | 57 | 62 |
|  | **Equivocal** | 5 | 1 | 6 |
|  | **Total** | 99 | 64 | 163 |

Concordance rate was 93.0%% between QDB_4B5_ and FISH results.

* Based on cutoff value at 0.399 nmole/g for 4B5.

^#^ Equivocal cases, including 2+ from IHC analysis, were not included in the comparison.
